# Postbiotics: The concept and their use in healthy populations

**DOI:** 10.3389/fnut.2022.1002213

**Published:** 2022-12-09

**Authors:** Gabriel Vinderola, Mary Ellen Sanders, Seppo Salminen, Hania Szajewska

**Affiliations:** ^1^Instituto de Lactología Industrial (CONICET-UNL), Faculty of Chemical Engineering, National University of Litoral, Santa Fe, Argentina; ^2^International Scientific Association for Probiotics and Prebiotics, Centennial, CO, United States; ^3^Functional Foods Forum, Faculty of Medicine, University of Turku, Turku, Finland; ^4^Department of Paediatrics, Medical University of Warsaw, Warsaw, Poland

**Keywords:** postbiotics, definitions, healthy population, microbiome, clinical studies

## Abstract

The term postbiotic was recently defined by an panel of scientists convened by the International Scientific Association of Probiotics and Prebiotics as “a *preparation of inanimate microorganisms and/or their components that confers a health benefit on the host.*” This definition focused on the progenitor microbial cell or cell fragments, not just metabolites, proteins or carbohydrates they might produce. Although such microbe-produced constituents may be functional ingredients of the preparation, they are not required to be present in a postbiotic according to this definition. In this context, terms previously used such as paraprobiotics, ghostbiotics, heat-inactivated probiotics, non-viable probiotics, cell fragments or cell lysates, among others, align with the term postbiotics as conceived by this definition. The applications of postbiotics to infant nutrition and pediatric and adult gastroenterology, mainly, are under development. Some applications for skin health are also underway. As postbiotics are composed of inanimate microorganisms, they cannot colonize the host. However, they can in theory modify the composition or functions of the host microbiota, although evidence for this is scarce. Clinical results are promising, but, overall, there is limited evidence for postbiotics in healthy populations. For example, postbiotics have been studied in fermented infant formulas. The regulation of the term postbiotic is still in its infancy, as no government or international agency around the world has yet incorporated this term in their regulation.

## Introduction

Usage of the term “postbiotic” began rapidly increasing in recent years. Ninety-three percent of all papers retrieved on PubMed using the term “postbiotic” have occurred since 2018 through present day (September 14, 2022). Over 50% of these articles were published since the International Scientific Association for Probiotics and Prebiotics (ISAPP) published a consensus panel on the term “postbiotic” (1). Although statistics are not available, the term postbiotic is also emerging on commercial products for humans and animals. Interest in this term is clearly growing and warrants considered discussion. The intention of this perspective article is to address a subset of postbiotic applications, specifically orally consumed postbiotics and evidence for their use in healthy populations. Therapeutic use is not considered herein, but was summarized previously (1). Further, this article discusses the extent to which postbiotic effects have been shown to be mediated by microbiome modulation. Finally, some perspectives on regulatory challenges are addressed, using the situation in the European Union as an example.

### Understanding the postbiotic definition and scope

In 2021, a consensus panel of experts convened by the International Scientific Association of Probiotics and Prebiotcis published a definition and scope for the term “postbiotic.” Six varied definitions had been published previously (1). The panel concluded that existing definitions were insufficient for this emerging field and proposed the definition “*preparation of inanimate microorganisms and/or their components that confers a health benefit on the host*.” The key element of a postbiotic is the presence of inanimate microbes, but other physiologically active microbial cellular components (such as cell wall fragments or enzymes) or metabolites can contribute substantively to the complexity and functionality of the postbiotic preparation. These components are not required of a postbiotic, but certainly reflect the broad scope of the preparation as defined. The panel deemed that purified metabolites and purified cell components do not fall under the scope of postbiotics. Microbe-derived substances, such as vitamins, organic acids such as butyric acid, antibiotics, to name a few, have specific chemical names of their own that can be used and if needed, can be referred to collectively as microbe-derived substances. Postbiotics are not limited to the gastrointestinal tract. They may be effective in other body sites such as the skin, the respiratory tract or the vagina.

Recognizing that the term “postbiotic” means “after life” (not “from life”), the postbiotic term seemed appropriately used for preparations of microbes prepared alive and then inactivated. Recent innovative examples of killed microbes having a benefit on health suggest the importance of this emerging area (2, 3), which are captured under the umbrella term, “postbiotic” as defined by ISAPP. Further, to the extent inanimate microbes have a physiological impact on the host, it raises important questions regarding how we perceive probiotics. Restricting “postbiotics” to metabolites would miss an opportunity to best capture these developments under one concept.

The ISAPP definition followed the principles applied to ISAPP consensus definitions of probiotics, prebiotics, and synbiotics. The definitions refer to consumed or applied substances, not to substances produced *in situ*; should be broad enough to support innovation; should not restrict host (not just humans), regulatory category (not just foods), or site of action (not just gut); and should allow for multiple mechanisms of action (for example, not just microbiota modulation or immunomodulation).

It had been proposed that a postbiotic must be derived from a probiotic (4). Since a probiotic must have a documented health effect, this stipulation would impose an unnecessary and expensive burden to prove a health benefit of the progenitor microbe used to make a postbiotic. Resources would better be dedicated to proving health benefits of the postbiotic.

## Postbiotics and the microbiome

Evidence for the effect of postbiotics on microbiota composition and function is scant. A preclinical, animal model was used to study a pure culture of heat-inactivated *Lacticaseibacillus paracasei* N1115 (not including its metabolites), being developed to protect neonates from harmful effects of antibiotics (5). This preparation mitigated antibiotic-induced changes in the composition of the intestinal microbiota. A substantial modification in the composition of the microbiota due to antibiotics administration was observed, whereas the administration of heat-inactivated *L. paracasei* N1115 was able to partially restore the gut microbiota disrupted by antibiotic exposure. A preparation of heat-inactivated *Bacillus subtilis* (strain not declared) and *Lactobacillus acidophilus* BFI tested in yellow-feathered broilers enhanced feed efficiency, and at the same time was able to induce a decrease in plasma contents of cholesterol and creatinine. These endpoints were associated with changes in the composition, diversity and functions of the cecal microbiota (6). The effect of the administration of a standard mouse chow containing a heat-killed fermentate produced by two *Lactobacillus* strains (strains identity not declared), on the behavior and microbiota of healthy mice was studied (7). Increased sociability and lower baseline corticosterone levels were observed followed prolonged consumption of these products, and also led to modest but significant changes in the microbiota. Differences in different bacterial genera were observed between control and treated mice. In treated mice, the relative abundance of *Alistipes* and *Odoribacter* was consistently reduced, while *Prevotella* was increased. Major differences in the composition of the microbiota between groups were not observed, but feeding the fermentate led to subtle modifications. These few studies in animals show small changes in microbiota composition, no data on function, and the importance of these changes is not evident.

Evidence of microbiome modulation by postbiotics in humans is limited. Stress-associated symptoms in healthy young adults and gut symptoms in patients with irritable bowel syndrome were improved by the administration of heat-inactivated *Lactobacillus gasseri* CP2305 (8). Intake of this inactivated strain significantly reduced anxiety and sleep disturbance relative to placebo. In addition, 16S rRNA gene sequencing of participant fecal samples showed attenuation of the stress-induced loss of *Bifidobacterium* spp. and the stress-induced increase of *Streptococcus* spp.

The role of fermented foods as postbiotics is an exciting area for research. The inanimate microbes, metabolites, and fibers delivered in some fermented foods have the potential to make a “package” of functional components that may be shown to enhance health as well as improve the microbiome. A potential postbiotic fermented food may be sourdough bread, a food with inanimate microorganisms, due to baking, and their metabolites, produced during fermentation, for which impact on gut microbiome and health benefits were reported (9). Health benefits were reported also for heat-inactivated fermented milks (10). Some fermented foods are heat-treated with the goal of increasing shelf life, even though such treatment kills viable fermentation microbes, can inactivate functional ingredients such as heat-sensitive vitamins and lactase, and may lead consumers to see the food as being less fresh. Nonetheless, such fermented foods could in the future be shown to deliver efficacious postbiotics.

When discussing the ability of postbiotics to alter the microbiome, it should be remembered that microbiome changes are not required to be demonstrated for a postbiotic, and indeed, this may not be the mechanism by which postbiotics deliver health benefits. For instance, heat-inactivated *Lactiplantibacillus plantarum* nF1 promoted intestinal health (improvements in fecal pellet number, weight, water content, intestinal transit length, and contractility) in rats with loperamide-induced constipation. No changes in microbiota composition compared to the control group were detected (11). Some postulated mechanisms of action for postbiotics beyond microbiota modulation include enhancement of epithelial barrier function, modulation of local and systemic immune responses, and/or systemic signaling *via* the nervous system (1). Mechanisms of action that require metabolic activity, such as the *in situ* inhibition of pathogens by metabolites produced by a live microbe, would not be functional in a postbiotic.

## Postbiotics in healthy population

In this section, recent developments are summarized on the safety and efficacy of postbiotics for use in healthy populations, which we defined as the absence of reported disease in included subjects. To obtain evidence, the Cochrane Central Register of Controlled Trials and MEDLINE databases were searched (although not systematically) in July 2022 for randomized controlled trials (RCTs) or meta-analyses that compared postbiotics with placebo or no therapy. Because in the past different terms have been used to refer to preparations that meet the current definition of postbiotic, comprehensive literature searches for “postbiotics” need to include other terms, such as non-viable probiotics, heat-inactivated probiotics, heat-treated probiotics, tyndallized probiotics, paraprobiotics, ghostbiotics, cell lysates or cell fragments.

### Postbiotics in children

#### Infant formulas with postbiotics

Infant formulas with postbiotics are those fermented with lactic acid–producing bacteria during the production process but not containing significant numbers of viable bacteria in the final product (12). Thus, previously such formulas were known as fermented formulas. In addition to the fermentation process, physical treatment, which may include homogenization, pasteurization, sterilization, and/or spray-drying, is often applied. Such formulas are increasingly available in many countries. A 2022 systematic review of RCTs (search date: December 2021) summarized evidence on the clinical efficacy and safety of the postbiotic infant formulas (with/out other modifications) (13), where eleven RCTs were included. Most studies evaluated infant formulas fermented with *Bifidobacterium* breve C50 and *Streptococcus thermophilus* 065. As previously reported (12, 14), such formulas, compared with non-supplemented infant formula, were safe and well-tolerated. Postbiotic formulas with additional compositional changes (including, formula fermented with *Bifidobacterium breve* C50 plus *S. thermophilus* 065, including prebiotics; formula partly fermented with *B. breve* C50 plus *S. thermophilus* 065, including prebiotics with or without modified milk fat; anti-regurgitation formula partly fermented with *B. breve* C50 plus *S. thermophilus* 065, including prebiotics, 3′-galactosyllactose, and 3′-GL) were also generally safe and well-tolerated. For non-clinical outcomes, reported as the primary outcomes, data were limited. Fermented formulas with *B. breve* C50 and *S. thermophilus* 065 reduced fecal pH values and increased fecal IgA levels. However, whether these changes *per se* are of benefit is not proven but cannot be excluded. For example, IgA plays an essential role in mucosal immunity; thus, it may impact the overall immunity of infants. There were insufficient data to conclude on health benefits of formula fermented with *L. paracasei* CBA L74.

#### Prevention of common infectious diseases

A 2020 systematic review (search date: March 2019) evaluated evidence on the use of postbiotics to prevent and treat common infectious diseases among children younger than 5 years (15). Seven RCTs involving a total of 1,740 children met the inclusion criteria. For preventive trials, the pooled results from two RCTs (*n* = 537) showed that, compared with the placebo, heat-inactivated *L. paracasei* CBA L74 reduced the risk of diarrhea [relative risk (RR) 0.51; 95% CI 0.37–0.71], pharyngitis (RR 0.31; 95% CI 0.12–0.83), and laryngitis (RR 0.44; 95% CI 0.29–0.67). Although outside of the scope of this paper, in therapeutic trials, compared with the placebo, supplementation with heat-killed *L. acidophilus* LB reduced the duration of diarrhea (4 RCTs; *n* = 224; mean difference, −20 h; 95% CI −27 to −13.5).

A 2020 RCT performed in 172 healthy children aged 3 to 6 years, found that the administration for 4 months of a heat-killed *Pediococcus acidilactici* K15 was not effective for preventing respiratory tract infections among preschool children. Compared with the placebo group, in the K15 group the salivary sIgA level in the K15 group was significantly higher (16).

A 2006 RCT investigated the effect of micronutrients (including zinc) with or without heat-inactivated *L. acidophilus* compared to a placebo in 75 infants aged 6–12 months who were at high risk for diarrhea-related mortality (defined as at least one episode of diarrhea in the preceding 2 weeks). The prevalence of diarrhea was 26% in the micronutrient with heat-inactivated *L. acidophilus* group, 15% in the micronutrient group, and 26% in the placebo group. The difference between the micronutrient with *L. acidophilus* group and placebo group was not significant (17).

### Postbiotics in adults

A RCT conducted in 280 healthy adults (> 65 years) found that the consumption for 20 weeks of heat-killed *Lactiplantibacillus pentosus* b240 in a low (2 × 10^9^ cfu) or high (2 × 10^10^ cfu) dose compared with placebo resulted in differences in accumulated incidence rate of the common cold (29.0 vs. 34.8 vs. 47.3% respectively; *p* for trend = 0.012). Quality of life measured by a validated questionnaire increased in both intervention groups (*p* for trend = 0.016) (18).

A small randomized, controlled trial performed in healthy individuals (aged 20–70 years) with a tendency toward constipation (*n* = 20) or frequent bowel movements (*n* = 19) found that the consumption for 3 weeks of *Lactobacillus gasseri* CP2305-fermented heat-treated milk compared with artificially acidified milk-based placebo beverage significantly improved Bristol stool scale scores (*p* < 0.05). Output and color tone were also improved, especially in subjects with a tendency toward constipation (18). Another trial from the same group, involving 118 healthy adults with relatively low or high stool frequencies, found that the administration for 3 weeks of the same test product of *L. gasseri* CP2305 compared with the placebo beverage had positive effects on the number of evacuations and the scores for fecal odors (*p* = 0.035 and *p* = 0.04, respectively). Both studies also evaluated the effects on certain microbiota parameters. However, the results were not consistent. For example, with regard to the content of *Clostridium* cluster IV, it was significantly increased in the first study (19) but decreased (*p* < 0.003) in the second study (20).

A RCT in 59 healthy subjects (18–65 years) found that the consumption (for 6 weeks) of juice containing live or heat-inactivated *L. rhamnosus* GG compared with control juice without live or heat-inactivated bacteria had no effect on human rhinovirus (HRV) load (*p* = 0.57). HRV load positively correlated with symptom scores (on days 2 and 5: *p* < 0.001 and *p* = 0.034, respectively) (21).

A large RCT involving almost 2,200 healthy adults, aged between 20 and 59 years, found that the consumption for 12 weeks of heat-killed *Levilactobacillus brevis* KB290 in combination with β-carotene did not significantly reduce influenza incidence, fever incidence, or incidence/degree of clinical symptoms. However, the study product significantly reduced influenza incidence in the subjects aged < 40 years (*n* = 1,077). No serious adverse events were reported (22).

A number of RCTs have evaluated the effects of various postbiotics on non-clinical outcomes. For example, heat-killed *L. gasseri* TMC0356 was found to enhance some aspects of cellular immunity in the elderly (23). Heat-killed *L. plantarum* L-137 was found to enhance innate immunity (type I interferon production) (24), as well as acquired immunity (especially Th1-related immune functions) in healthy adults (25). Moreover, daily intake of heat-killed *L. plantarum* L-137 improved biomarkers of lipid metabolism and inflammatory mediators in overweight but otherwise healthy adults (26). It is undetermined if these changes in non-clinical parameters translate into clinical benefits.

## Challenges for conducting studies on postbiotics in healthy populations

Considering the evidence discussed above, rarely are there data from more than single studies on a given postbiotic. Factors that inhibit repetition of trials include lack of interest due to absence of scientific novelty and/or difficulty in securing funds to conduct a confirmation trial. Commercial sponsors may not be interested in confirming a positive result in cases of a postbiotic product available on the market. However, confirmatory studies provide confidence that initial observations are robust.

For this paper, the risk of bias in included studies was not formally assessed. However, some of the studies discussed had methodological limitations (i.e., unclear or inadequate randomization and/or allocation concealment, blinding, and/or intention-to-treat analysis). Only for some of the included trials were sample size calculations available.

The trials also varied in outcomes assessed. The lack of core outcome sets (defined as an agreed-upon standardized set of the most important outcomes), specific for each condition, which include both benefits and harms and are relevant within routine clinical practice is an important unmet need, and makes comparison of the studies, even addressing the same clinical problem, difficult. However, critically relevant outcomes that inform the value of consumption of postbiotics in healthy populations may take years to become apparent. For example, in a pediatric population if the outcome of interest is prevention of allergic diseases, a few years’ follow-up is needed.

Finally, heterogeneity of available evidence for postbiotic effects in healthy populations can be seen in the presence in the intervention of other potentially active ingredients (such as the addition of prebiotics in some infant formulas trials) and varied timing and length of interventions. Heterogeneity makes it difficult to combine results from different trials to lead to an overall conclusion on efficacy. Further, while there is a rationale for performing studies on combination products, such studies do not allow conclusion about which ingredient in a complex formulation contributed to any observed overall effect.

## Challenges for the regulation of the term postbiotics: The European Union example

Regulatory frameworks for foods generally encompass guidance for establishing safety for use by the general population and specifics about health benefit claims that are allowable to be made on a product. The absence of a consensus definition for postbiotics has hindered development of regulatory frameworks for probiotics due to lack of guidelines in the area. The European safety assessment of microbes in food is based focused on use of live microbes, using the unique Qualified Presumption of Safety (QPS) list, a systematic collection of safety data on microbes and after evaluation placement on the QPS list.^1^ To make the QPS list, the evidence of history of safe use at the species level and exposure to humans in food is sufficient to conclude about safety. Evaluation at the strain level is needed to document an antibiotic resistance profile that minimizes a risk of horizontal transfer of antibiotic resistance genes to host microbes. If so, then the regulatory pathway is easier as no safety concern is usually presented with them. The QPS list and evaluations are always published in the European Food Safety Authority (EFSA) journal, where background for either inclusion in the list is given or reasons for not accepting the microbe to the QPS list are discussed. The list is updated annually by the EFSA Biohazards Panel (see text footnote 1).

In the European Union, postbiotics, similar to probiotics and prebiotics, face both novel food regulation and health claim regulation. If the microbe, being probiotic or postbiotic, falls into the novel food category, the safety evaluation is more exhaustive and requires toxicological information among other safety requirements (27, 28). The EFSA guidance document in the case of safety assessment of live microbes is challenging and lacks clear guidelines. Recent experience demonstrates that safety of inanimate bacteria (potential postbiotics) may be easier to achieve than safety of live bacteria. Safety assessment of inanimate bacteria as novel food is now available for three different preparations (*Bacteroides xylanisolvens, Akkermansia muciniphila*, and *Mycobacterium sentence manresensis*) [(29–31), respectively]. These serve as a models for requirements and all include the means of inactivation of the live microbes. These three preparations have not been accepted for QPS status as live preparations. *Bacteroides xylanisolvens* and *Akkermansia muciniphila* were assessed positively for use as inanimate novel food by EFSA and following EU Commission authorization. However, unlike the other inanimate microbes assessed as safe novel foods, *Mycobacterium* has not been authorized by the European Commission.

Taken together, it appears easier to fulfill regulatory requirements for postbiotics (inanimate microorganisms) compared to probiotics (live microorganisms), even though to our knowledge, no regulators have yet taken a position on the postbiotic definition. The consensus definition discussed herein is therefore especially timely and relevant for postbiotic formulations for food or pharmaceutical applications.

In the European Union, no specific regulation covers postbiotics, but since their consensus definition requires a demonstrated health benefit, current interpretation is that the use of the terms on a food or food supplement would require health claim approval by EFSA and systematic novel food application and approval in Europe before the term can be used in foods or feeds. The recent EU Regulation (EU) 2017/745 for medical devices also has a specific paragraph positioning “living organisms” out of the scope of the Regulation.

## Conclusion

Looking to the future, it is important that the scientific field coalesces around a given definition of postbiotic. ISAPP has proposed a definition that is in line with the other “biotic” definitions and is clearly stated. The field lacks evidence regarding the ability of postbiotics to alter the gut microbiome, but like probiotics, postbiotics may induce health benefit by mechanisms other than microbiome modulation. Overall, there is growing evidence that postbiotics may have benefits for healthy pediatric and adult populations, these promising results require confirmatory studies to make further nutritional recommendations. Both positive and negative (null) studies have been published. As yet, no clear recommendations for or against the use of postbiotics in healthy population can be formulated. As not all interventions within the group of postbiotics are equal, safety, the capacity to alter the microbiome and efficacy should be established individually for each specific intervention.

## Data availability statement

The original contributions presented in this study are included in the article/supplementary material, further inquiries can be directed to the corresponding author.

## Author contributions

MS described the definition of postbiotics and discussed the limitation of previous definitions. GV covered the issue of postbiotics to modifying the structure, function or functionality of the gut microbiota. HS covered the topic related to postbiotic in healthy populations and the challenges of conducting clinical studies. SS covered the regulatory aspects of postbiotics. All authors contributed to the article and approved the submitted version.

## Footnotes

1
https://www.efsa.europa.eu/en/topics/topic/qualified-presumption-safety-qps
